# Chronic polypharmacy impairs explorative behavior and reduces synaptic functions in young adult mice

**DOI:** 10.18632/aging.103315

**Published:** 2020-05-22

**Authors:** Francesca Eroli, Kristina Johnell, María Latorre Leal, Chiara Adamo, Sarah Hilmer, Jonas W. Wastesson, Angel Cedazo-Minguez, Silvia Maioli

**Affiliations:** 1 Karolinska Institutet, Department of Neurobiology, Care Sciences and Society, Center for Alzheimer Research, Division of Neurogeriatrics, Solna, Sweden; 2 Department of Medical Epidemiology and Biostatistics, Karolinska Institutet, Stockholm, Sweden; 3 Kolling Institute, Royal North Shore Hosptial and University of Sydney, Clinical Pharmacology and Aged Care, Sidney, Australia; 4 Aging Research Center, Karolinska Institutet and Stockholm University, Stockholm, Sweden

**Keywords:** polypharmacy, adverse outcomes, memory, behavior, synaptic proteins

## Abstract

A major challenge in the health care system is the lack of knowledge about the possible harmful effects of multiple drug treatments in old age. The present study aims to characterize a mouse model of polypharmacy, in order to investigate whether long-term exposure to multiple drugs could lead to adverse outcomes. To this purpose we selected five drugs from the ten most commonly used by older adults in Sweden (metoprolol, paracetamol, aspirin, simvastatin and citalopram). Five-month-old wild type male mice were fed for eight weeks with control or polypharmacy diet. We report for the first time that young adult polypharmacy-treated mice showed a significant decrease in exploration and spatial working memory compared to the control group. This memory impairment was further supported by a significant reduction of synaptic proteins in the hippocampus of treated mice. These novel results suggest that already at young adult age, use of polypharmacy affects explorative behavior and synaptic functions. This study underlines the importance of investigating the potentially negative outcomes from concomitant administration of different drugs, which have been poorly explored until now. The mouse model proposed here has translatable findings and can be applied as a useful tool for future studies on polypharmacy.

## INTRODUCTION

Older people represent a growing proportion of the population [1] and are the most frequent users of medications [2–5]. Polypharmacy, defined as the concomitant use of five or more drugs [6], is very common in old age [7].

Due to the presence of different parallel health conditions and impairments (e.g. cognitive deficits, renal failure, orthostatic hypotension and gastrointestinal disorders), older adults are more likely to suffer from adverse drug effects than younger populations [8]. Moreover, age-related changes in pharmacodynamics and pharmacokinetics contribute to stronger effects of many drugs and therefore the risk of negative outcomes [9]. In epidemiological studies, polypharmacy has been associated with enhanced risks of adverse events in older people which comprise falls, hospitalization and higher mortality [10–13]. Increased chances of developing cognitive disturbances and decline have also been linked with inappropriate use of multiple medications in older adults [14–16]. Despite evidence from observational studies, little is known about the harmful effects of multiple-drug therapy in older people in experimental studies [17], as most of the studies on animals and humans focus on the use of drugs as monotherapy, and randomized clinical trials often exclude older patients [18].

A typical approach when studying the adverse effects of pharmacological compounds at the preclinical level is to use animal models [19, 20]. Previous studies have successfully translated findings from mice to humans, and this is especially evident in the investigation of mechanisms behind potential side effects. The obtained information can then be validated in human studies such as biomarker correlation analysis and clinical trials.

To our knowledge, only two preclinical studies on the effects of polypharmacy have been conducted until now. In the first study it was shown that short-term administration (two to four weeks) of five medications reduced physical functions in old but not young wild-type mice [21], while the second one tested chronic administration (12 months) of polypharmacy with different drug burden indices in aged mice, reporting higher frailty, impaired mobility and functional activities, which were reversible [22]. Since chronic exposure to polypharmacy is very common in older adults [23], the present study aims to deepen the experimental knowledge on polypharmacy by further investigating whether long-term exposure (four to eight weeks) to a multiple-drug treatment could lead to any adverse events including cognitive effects in young adult mice. Toward this aim, we have fed wild-type mice with a chronic polypharmacy diet. The polypharmacy regimen consisted of five pharmaceutical compounds that were chosen among the ten most commonly used medications in older people in Sweden [24]: metoprolol (a β-blocker used to treat angina and hypertension), paracetamol (acetaminophen, a widely used analgesic), low-dose aspirin (as a blood thinning for the prevention of cardiovascular disease), simvastatin (a statin used to lower blood cholesterol and triglycerides), and citalopram (an antidepressant belonging to the selective serotonin reuptake inhibitor class). Food intake, body weight, serum creatinine, albumin and alanine aminotransferase (ALT) levels were measured as basic variables for health status, while functional and psychological outcomes, including locomotor activity and function, anxiety-like behavior, spatial and non-spatial memory were assessed by behavioral tests. Finally, biochemical analyses were performed on hippocampus of treated mice to investigate possible changes in synaptic proteins that could account for the behavioral phenotype observed in polypharmacy treated mice.

## RESULTS

### Body weight and food intake

**Figure 1:**
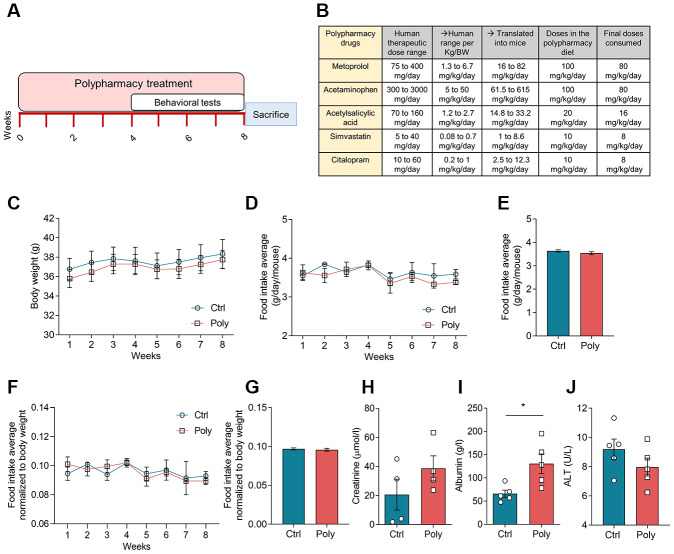
**Effects of polypharmacy treatment on body weight and food intake, and serum protein levels.** (**A**) The diagram above outlines the timeline of the polypharmacy study. (**B**) The table indicates the composition of the polypharmacy diet and drug dosages, specifying the human therapeutic range per person and per Kg/body weight (BW) and translated into mice. (**C**) The curves show mouse body weight average monitored over the eight weeks of treatment. (**D**–**G**) The curve and histogram plots express the FI as weekly and total average over the study period, measured as real values (**D**, **E**) or normalized to body weight (**F**, **G**). Animals per group: n= 9 control group, n= 10 polypharmacy group. (**H**–**J**) Histograms indicate serum creatinine, albumin and ALT levels; creatinine: n= 4, 4; albumin: n= 5, 5: ALT: n= 5, 5, for control and polypharmacy groups respectively; *p<0.05, Mann-Whitney test. All data are presented as mean ± SEM. Ctrl= control, Poly= polypharmacy.

The polypharmacy treatment was well-tolerated and no signs of illness or mortality among the mice were observed over the study period. During the eight weeks of treatment, all the mice gradually increased in body weight (Figure 1C), as normal for young adult animals. There was no significant difference in body weight between control and polypharmacy group. Food intake (FI) average was also checked weekly without any significant difference between groups. FI was measured as weekly average (Figure 1D) and as total average over the study period (Figure 1E); moreover, we performed the same analysis normalized to the mouse body weight (Figure 1F, 1G). Mice consumed about 20% less than the anticipated FI and the final doses of administered drugs were: 80 mg/Kg/day metoprolol, 80 mg/Kg/day acetaminophen, 16 mg/Kg/day acetylsalicylic acid, 8 mg/Kg/day simvastatin and 8 mg/Kg/day citalopram. These final drug concentrations result to be within the therapeutic dose range in humans, as reported in the table in Figure 1B. The table shows human therapeutic dose range intervals per each compound and the corresponding interval translated into mice, followed by the final doses consumed by the animals (calculation of human-to-mouse dose translations is explained in the methods).

Serum creatinine and albumin were measured at the end of the treatment period to monitor renal and hepatic functions. Creatinine levels did not change significantly in polypharmacy mice compared to controls (control: 20.6±10.5 μmol/l, n=4; polypharmacy: 38.7±8.8 μmol/l, n=4; Figure 1H) while albumin levels were found to be significantly higher in polypharmacy fed mice (control: 66±7.1 g/l, n=5; polypharmacy: 130.3±20.8 g/l, n=5; *p<0.05, Mann-Whitney test; Figure 1I). Serum ALT was measured at the time point corresponding to half of the treatment (4 weeks), as a further indicator of hepatic injury or fatigue. There were no significant changes in ALT levels of polypharmacy mice compared to controls (control: 9.2±0.7 U/L, n=5; polypharmacy: 8.0±0.6 U/L, n=5; Figure 1J).

### Locomotor activity and anxiety-like behavior: polypharmacy diet affects exploratory pattern

**Figure 2:**
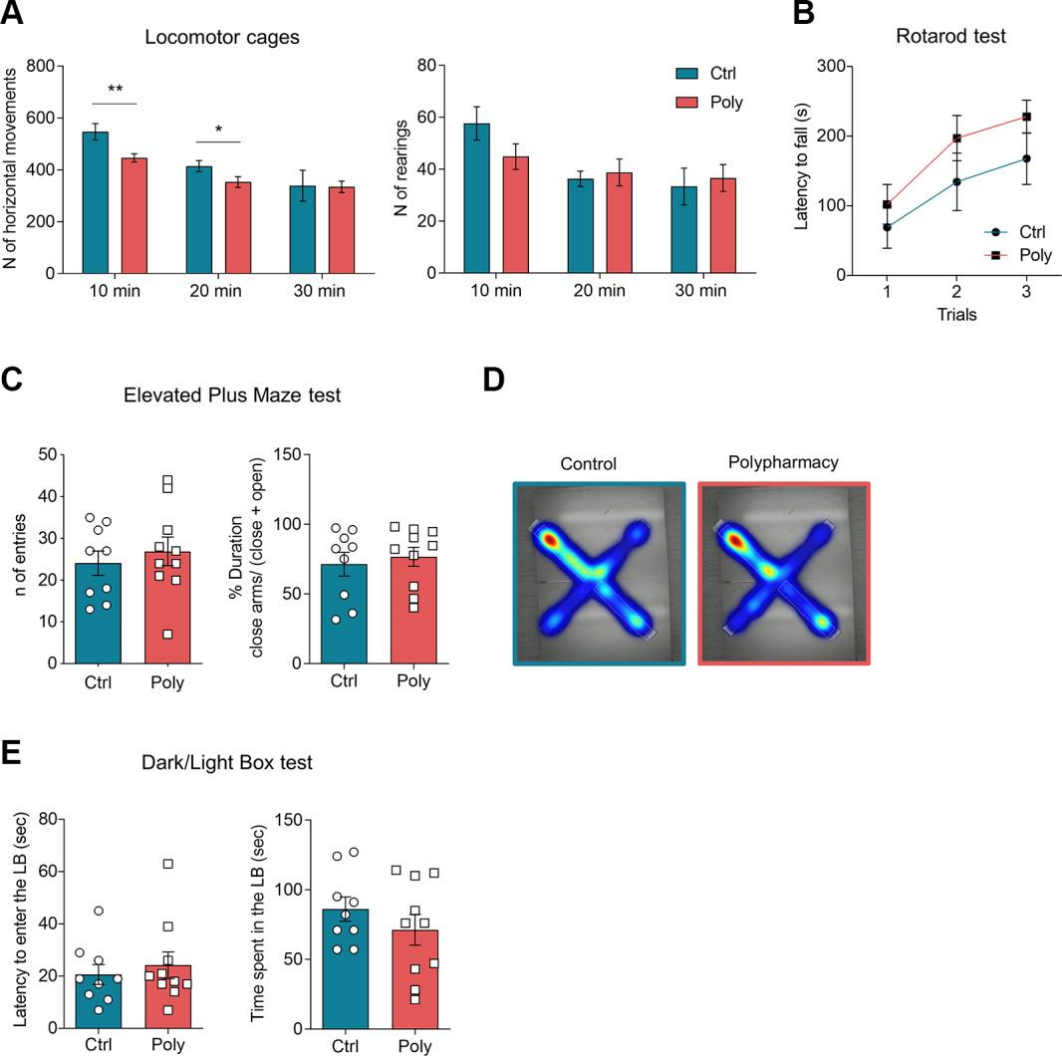
**Locomotor activity and anxiety-like behavior in control and polypharmacy treated mice.** (**A**) Locomotor activity and explorative behavior: histograms show horizontal and vertical (rearing) activity measured in OF cages and analyzed per time intervals of 10 minutes over a total duration of 30 minutes. Interaction between time and treatment groups were analyzed with two-way ANOVA repeated measurements test; *p≤0.05, **p<0.01. (**B**) Rotarod test: average of latency to fall measured during the 3-trial session. (**C**, **D**) EPM test: dot plots show the number of entries and percentage of time spent in closed arms. Heatmaps represent color-coded areas of the maze, where red zones indicate the area which the mice explored the most (maps are showed as control and polypharmacy group average). (**E**) DLB test: dot plots display first latency to enter the LB and time spent by the mice exploring that compartment. All data are presented as mean ± SEM. Animals per group: n= 9 control, 10 polypharmacy. Ctrl= control, Poly= polypharmacy.

To assess general locomotor activity mice were tested in Open Field (OF) cages. Horizontal and vertical activity were first analyzed over the total 30-minutes trial duration and then in time intervals of 10 minutes, in order to evaluate the habituation and the next phases of explorative behavior [25–27]. From the analysis of rearing behavior, no significant difference was observed between the two groups both for the total activity (123.7 ± 8.1 units in control vs 116.7 ± 13.0 units in polypharmacy mice, data not shown) and for the 10-minutes interval analysis (Figure 2A, right histograms). Nevertheless, 10-minutes interval analysis of horizontal activity showed a significantly decreased exploratory behavior in polypharmacy mice when compared to controls (Figure 2A left plot; two-way ANOVA repeated measurements test, *p≤0.05, **p<0.01), while they did not exhibit significant changes in the total horizontal activity (1262.6 ± 76.2 units in control vs 1099.9 ± 57.0 units in polypharmacy mice, data not shown). Motor coordination and balance were evaluated through Rotarod test and both control and treated animals showed similar performances (Figure 2B).

Polypharmacy treatment did not affect anxiety-like behavior investigated through Dark/Light Box (DLB) and Elevated Plus Maze (EPM) experiments: in the EPM test control and polypharmacy mice performed a comparable number of entries and both showed to prefer the enclosed arms (see histograms and heatmaps in Figure 2C and 2D). Likewise, LDB test showed that mice administered polypharmacy diet exhibited similar latency to enter the LB and time spent in that compartment than control diet fed ones as illustrated in Figure 2E.

### Effect of polypharmacy regimen on memory and learning

**Figure 3:**
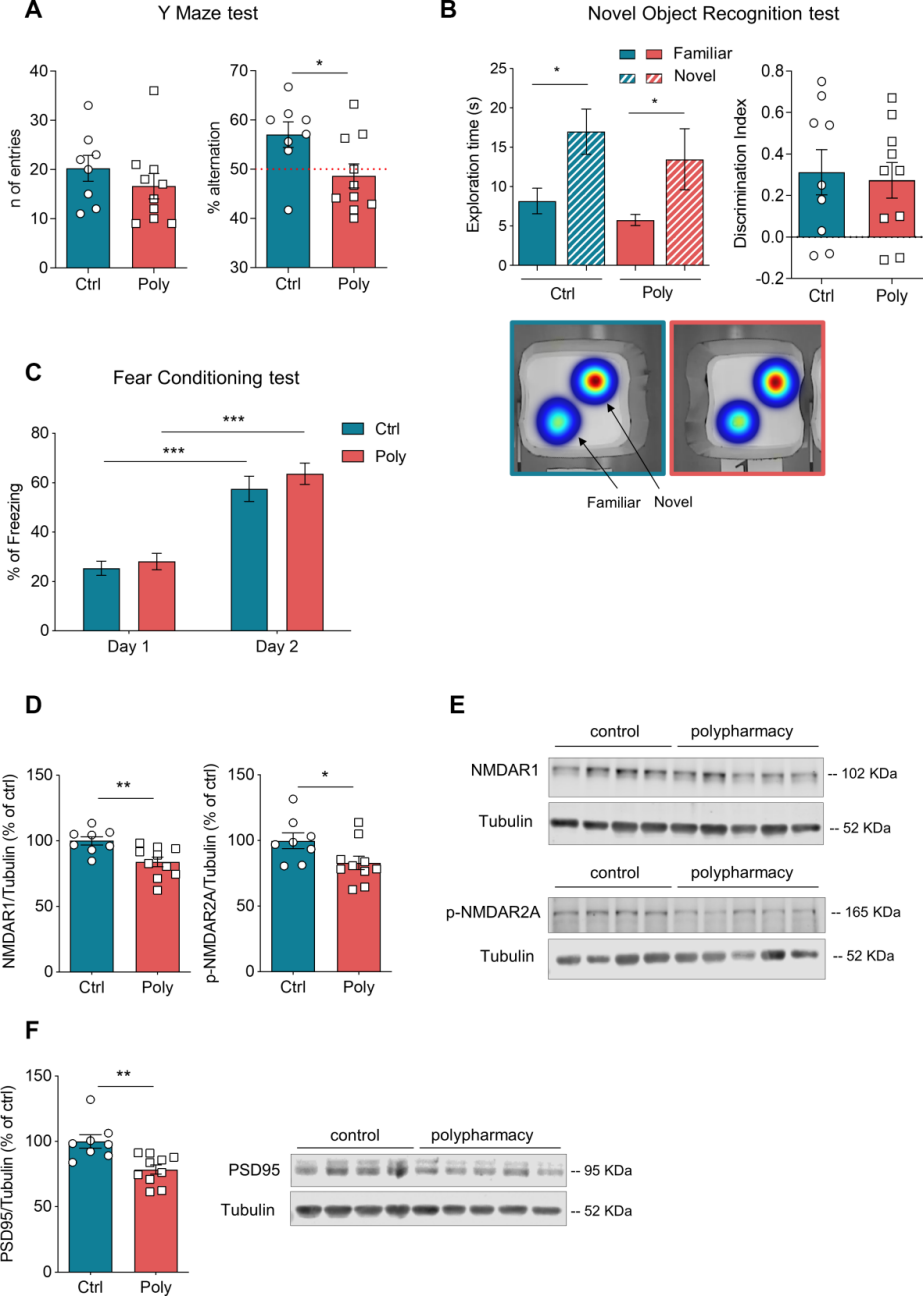
**Memory tests and immunoblotting analysis: postsynaptic protein levels were decreased in hippocampus of polypharmacy fed mice.** (**A**) Y maze test: graphs indicate the number of entries and percentage of spontaneous alternations performed by control and polypharmacy mice. Note that while 90% of control animals alternated above the 50% chance level, only 30% of polypharmacy mice did so, thus indicating a possible impairment in spatial working memory. *p<0.05, t-Student test. (**B**) NOR test (day 3, test day): histograms on the left show average of exploration time spent on the two objects, for control and polypharmacy group, *p<0.05; the statistical comparison between time spent on novel object vs familiar in control or polypharmacy group was analyzed with paired t-Student test. The respective heatmaps at the bottom visually represent the area explored around the objects, highlighting a clear preference for the novel object in both the groups (in red color the most visited zones). Dot graph shows the calculated discrimination index, where a score above 0 indicates that animals spent more time exploring the novel object than the familiar one. (**C**) Contextual FC test: percentage of freezing time measured on day 1 (habituation phase) and day 2 (context testing). ***p<0.001, two-way ANOVA repeated measurements test. (**D**–**F**) Hippocampal tissue samples from control and polypharmacy mice were analyzed by western blotting experiments. Dot plots show quantification of NMDAR1, phospho-NMDAR2A and PSD95 protein levels, which were significantly decreased in polypharmacy mice compared to control animals; *p<0.05, **p<0.01; t-Student test. Total protein levels were normalized with respect to α tubulin. On the right, representative examples of immunoblots for NMDAR1, phospho-NMDAR2A and PSD95 proteins. All data are presented as mean ± SEM. Animals per group: n= 9 control, 10 polypharmacy. Ctrl= control, Poly= polypharmacy.

Spatial working memory was assessed through Y maze test and the outcomes are shown in Figure 3A. No significant difference was detected among the two groups in the number of entries per arm (Figure 3A, left plot), whereas the analysis of spontaneous alternations showed that polypharmacy mice performed a significantly lower percentage of alternations compared to controls (right dot plot in Figure 3A, *p<0.05, t-Student test). Furthermore, we ran a one-sample t test to compare the alternation percentage of each group to the theoretical value of 50% chance level [28]: while control diet fed mice significantly alternated above the 50% chance level (p<0.05), polypharmacy fed mice did not, as only 30% of them alternated at levels above chance. These results suggest that polypharmacy treatment induced impairment in spatial working memory. The Novel Object Recognition (NOR) test was performed to evaluate non-spatial working memory. On day 3, both controls and treated animals were able to discriminate between the familiar and the novel object with a significant preference for the novel one, as shown by histograms of exploratory time and heatmaps in Figure 3B (red color zones indicates the area which they explored the most). The dot plot of discrimination index (Figure 3B) also indicates as most of the animals got a positive score, meaning more time spent on the novel object and thus the ability to recognize the novelty [29]. Figure 3C shows the outcomes of contextual Fear Conditioning (FC) test. During day 2, both control and polypharmacy mice exhibited a significantly increased percentage of freezing compared to day 1, indicating that they were able to learn and remember the association between the context and the negative stimulus (the foot shock). No differences were observed between the two groups suggesting that the polypharmacy diet did not affect contextual memory and learning.

### Decreased levels of synaptic proteins were found in the hippocampus of polypharmacy fed mice

To investigate any biochemical changes in polypharmacy mice, we ran western blotting experiments and measured the levels of synaptic proteins in the hippocampus. We analyzed postsynaptic proteins such as NMDA receptors (subunits NMDAR1 and phospho-NMDAR2A), which are known to be fundamental in activity-dependent synaptic plasticity and in learning and memory [30]. Figure 3D, 3E shows that levels of NMDAR1 and phospho-NMDAR2A were significantly lower in the hippocampus of polypharmacy mice compared to controls (NMDAR1: **p<0.01; phospho-NMDAR2A: *p<0.05; t-Student test; n= 9 control, 10 polypharmacy). We next analyzed the levels of PSD95, a scaffolding protein which is found in excitatory synapses of postsynaptic density complex and is involved in regulation of synaptic strength and transmission [31]. Immunoblot analysis showed in Figure 3F revealed that PSD95 levels were also significantly decreased in treated animals compared to controls (**p<0.01, t-Student test; n= 9 control, 10 polypharmacy).

## DISCUSSION

In the present study we characterized a mouse model of polypharmacy to explore potential harmful effects of long-term multiple drug treatment. We used young adult C57BL/6J male mice to establish whether concomitant administration of multiple medicines could affect locomotor activity, anxiety-like behavior, spatial and non-spatial or contextual memory already at young adult age. C57BL/6J is one of the most widely used mouse strains in preclinic pharmacology and toxicology studies [32, 33]. The choice of the animal strain is of particular importance when performing drug investigations and previous studies have indeed reported as different strains respond more or less effectively to the same compound [34, 35], depending on several variables like different pharmacodynamics and drug metabolism. For instance Jin ZL et al [34] found that some murine strains do not respond to citalopram to the same extent and in a dose-dependent way. We used citalopram at 10 mg/kg/day that is equivalent to a daily dose of 50 mg in humans, therefore close to its highest dosage in the range for treating depression (10–60 mg/day) [36, 37].

Polypharmacy diet was well-tolerated by the mice over the eight weeks of treatment and no differences in body weight or FI were observed. The analysis of serum ALT, creatinine and albumin resulted in increased levels of the latter in treated mice compared to controls. Increase of albumin in blood can be observed after a chronic treatment with multiple drugs, in particular, statins have been reported to prevent cardiovascular disease through the increment of human serum albumin levels [38]. Furthermore, paracetamol ability to bind serum proteins can lead to enhanced albumin concentration in the blood [39].

When the groups of mice were assessed for behavior, we observed for the first time that the concomitant use of different medications is able to affect critical functions at young adult age. We found that chronic administration of polypharmacy induced impairments in explorative behavior measured as reduced horizontal movements during OF test. When exposed to a new environment (such as the OF arena), rodents are willing to explore the novel area by innate behavior. A decrease in exploration reveals lack of curiosity and investigatory behavior which in healthy animals should be spontaneously present [40]. That can correlate to several variables as increased fear/anxiety or emotional factors [41] but can also be independent from anxiety-related components [42]. Moreover, we are the first to show that chronic exposure to five different medications affects spatial working memory, as emerged from the Y maze test, already in young adult mice. The significant deficit observed in the spatial memory test was further supported by the analysis of postsynaptic proteins in the hippocampus, which were found to be significantly decreased in treated mice. NMDA receptors and PSD95 complex are key contributors in synaptic plasticity and important mediators of many forms of memory and learning [30, 43]. Such experimental findings are novel and highly support epidemiological studies reporting that polypharmacy increases the probability of developing cognitive decline and disturbances in older adults [14, 16]. This suggests that our mouse model of polypharmacy has translatable findings and more in-depth studies are needed to investigate the possible mechanisms behind the reduction of synaptic proteins induced by polypharmacy.

The multiple-medication regimen did not significantly affect locomotor functions and coordination assessed with total OF analysis and Rotarod test, respectively. Likewise, anxiety-like behavior experiments (EPM and DLB tests) did not point out differences between polypharmacy and control groups and similar performances were also observed when testing the mice for non-spatial and contextual memory.

Huizer-Pajkos et al. [21] previously found that short-term exposure to multiple drugs led to locomotor impairments in old C57BL/6 male mice but not in young animals, suggesting that the 2-4 weeks intervention could be too short to fully observe the functional outcomes of polypharmacy in both young and old age. Mach et al. observed impairments in physical function after 12 weeks of treatment in middle age, as well as after 12 months of treatment in old age. Moreover, it is relevant to study the effects of chronic polypharmacy since older adults tend to have long-term exposure to multiple medications [23]. Our results suggest that chronic multiple compound treatment can impair exploratory pattern and cognitive function already at young adult age. These observations in young adult mice imply the importance for further investigating the adverse effects of multiple-drug regimens in old animals.

Most of the knowledge about drug-related adverse effects comes from studies on single-medication use. In the literature, monotherapy of the drugs composing our polypharmacy treatment has not been reported to be toxic [21, 44–48] or cause functional impairment in rodent models, when investigated- [22, 49, 50]. Regarding the effects of the individual drugs in the CNS, chronic citalopram monotherapy have been reported to decrease locomotor activity and grip strength in old mice [22]. Two studies performed in mouse models of Alzheimer´s disease have reported that citalopram and low-dose aspirin improves deficits in memory and learning [36, 51]. Paradoxical effects of paracetamol have been observed in rodent brain, showing that low doses of acetaminophen could have protective effects by reducing the oxidative stress status, while higher doses could lead to toxic effects on neurons and astroglia [52, 53]. The possible action of simvastatin, and statins in general, in the brain is still unclear: different clinical trials on patients reported conflicting results as some suggested impairing effects on cognition while other proposed beneficial ones [54]. Two preclinical research studies on murine models reported a beneficial effect of simvastatin against depression-like behavior and on memory and learning [49, 55], while another study observed that chronic simvastatin inhibited oligodendrocyte remyelination in mouse CNS thus affecting brain tissue repairing processes [56]. Metoprolol might cause minor dose-dependent side effects on CNS like slight depressed mind or disturbed sleep, although at therapeutic doses it should not affect the qualitative functions of the brain [57, 58]. A more recent investigation on the role of β-adrenoreceptors in the hippocampus has pointed out the importance of β-receptors 1 and 2 in memory consolidation, reporting that the blockade of β1-receptor by metoprolol impaired contextual and spatial memory in rats in FC and Morris water maze tests [59]. Taken together these aspects underline the importance of investigating the potentially harmful effects from concomitant administration of different drugs, which so far have been poorly explored. The approach and outcomes of our study can be applied in old mice to mimic the real-world setting where older adults frequently use multiple drugs [60]. The results from the present study can be valuable to interpret future results on aged mice, although the experimental design used here would need optimization due to possible age-related limitations (e.g. immobility of old mice, less reactivity to new stimuli).

In conclusion, this preclinical polypharmacy mouse model of five of the most commonly used drugs in the aged population in Sweden provides a proof of concept on harmful effects caused by multiple-drug administration. The use of rodent models to investigate drug development and adverse effects and to perform preclinical trials is a powerful tool which has been successfully applied to translate information to humans [61–63]. Therefore, the model we present here can be applied for future studies in aged mouse models to explore whether old age increases susceptibility to any adverse outcomes of polypharmacy. Species differences have been pointed out by some reports, highlighting differences in drug metabolism between mice and humans [64]. To overcome possible limitations related to this, future studies involving mouse strains humanized for certain drug-metabolizing enzymes [65] may be necessary. Sex differences in pharmacokinetics, pharmacodynamics and ageing should be addressed in future studies. Further experiments should address the exact mechanisms by which these adverse events occurred, as well the possibility of avoiding them by adjustments in doses or substitution of one or more drugs of the polypharmacy regimen. Some of the observed outcomes may be specifically related to the selected medicine combination. Therefore, it is important to evaluate and compare the results from different polypharmacy therapies with the final aim to design safer multiple-drug treatments in old age. In sum, the mouse model proposed here can be a useful tool to further investigate the potential negative outcomes of multiple medications.

## MATERIALS AND METHODS

### Animals

Wild-type C57BL/6J male mice were used for this study. Animals were purchased from Janvier Labs (France) at the age of 2 months and then housed in the animal facility in group of four-five mice per cage. (Karolinska Institutet, Huddinge, Sweden) in standard local conditions, with 12-h light/dark cycle, ad libitum access to food and water and cardboard tunnels and tissue paper as enrichment. At the age of 5 months they were randomly assigned to 2 different groups: control (n= 9) and polypharmacy (n= 10). Control mice were fed with a standard rodent diet (control diet) containing: 18.5% proteins, 5.5% oils and fats, 4.5% fiber (Teklad 2918 diet, Research Diet Inc., NJ, USA); the same diet base was used for the polypharmacy group, with the addition of drugs (polypharmacy diet).

Before starting the treatment, all mice were administered the control diet for a habituation period of 10 days, and thereafter they were randomly divided in two groups and fed with the control or the polypharmacy diet. After four weeks of treatment, at 6 months of age, the animals were tested for behavioral studies, while continuing the polypharmacy diet for other four weeks (see scheme in Figure 1A); the total duration of the treatment was eight weeks. Mice were weighed before starting the polypharmacy regimen and both body weight and FI (g food/mouse/day) were measured on weekly basis. Food was replaced every week with new food. After completing the behavioral testing, mice were sacrificed by cervical dislocation and trunk blood was collected. Brains were dissected and hippocampus and cortex were isolated and collected. Brain tissues were snap frozen in dry ice and immediately stored at -80 C until further use.

### Polypharmacy treatment

The polypharmacy treatment was selected based on the most commonly used medicines in older people in Sweden [24] and consisted of the following pharmaceutical compounds: metoprolol (100 mg/Kg/day; Sigma-Aldrich, USA) [66], paracetamol (100 mg/Kg/day; Sigma-Aldrich, USA) [67], acetylsalicylic acid (20 mg/Kg/day; Sigma-Aldrich, USA) [48], simvastatin (10 mg/Kg/day; Selleck Chemicals, USA) [68] and citalopram (10 mg/Kg/day; Selleck Chemicals, USA) [36]. Drug dosages per Kg/body weight in mice are considered to be 8- to 12.3- fold higher than in humans due to pharmacokinetic and pharmacodynamic differences [69, 70]. Based on this principle, and in accordance with previous studies on these drugs (cited above) as monotherapy in mice, polypharmacy compound doses were translated proportionally from humans to mice.

Estimated drug doses for the multiple-medication diet were calculated so to stay within the human therapeutic dose range and according to previous studies in rodents, where they were not found to be toxic when used alone [21, 44–48]. The table in Figure 1B indicates the daily therapeutic dose range in humans per person (second column) and per Kg/body weight (third column) considering an average of weight of 60-Kg in adults. Also, compound concentrations were chosen towards the higher dosage in the therapeutic dose interval, taking into account possible variations in the real FI compared to the estimated one. In the case of paracetamol we selected the lower dosage within the therapeutic dose range, due to its potential hepatotoxicity at high doses as reported in rodent studies [71, 72]. Final doses consumed by the animals were calculated afterwards depending on the actual FI observed. The concentration of compounds in the diet was calculated based on a FI of 0.17 ± 0.02 g food/g mouse/day according to previously observed FI for C57BL/6J mouse strain in our animal facility and literature [73].

### Ethical statement

All experimental procedures were performed in accordance with the local national animal care and use guidelines and approved by the local committee of Karolinska Institutet and the Swedish Board of Agriculture (ethical permit ID 827). All possible efforts were made to minimize any suffering or distress to the animals.

### Behavioral tests

At 6 months of age, the mice were tested for the following behavioral tasks: Open Field (OF), Elevated Plus Maze (EPM), Y Maze, Dark/Light Box (DLB), Rotarod, Novel Object Recognition (NOR) and Fear Conditioning (FC). The order of the different tests was chosen based on the level of stress caused by the procedure, starting from the less stressful [74]. A recovery time from one to six days was allowed after those tests considered more stressful or where sustained physical effort was required. All the tests were run between 9:00 and 14:00, by a female researcher. All the experiments were performed in white light. Prior to start each test, mice were brought to the experimental room for 45 minutes to acclimatize with the new environment.

### OF locomotor cages

The locomotor cages consisted of 35 x 35 cm square arenas. At the beginning of each trial the mouse was placed in the center of the arena and left free to explore for 30 minutes. The horizontal (i.e. walking) and vertical (i.e. jumping, rearing and grooming) activity was measured by infrared beams and photoreceptor cells, and recorded through MAD software (Nixus AB, Tumba, Stockholm). Before starting and between every trial the OF cages were cleaned with 70% ethanol solution.

### EPM test

The EPM apparatus consisted of two closed arms (30 x 5 cm, with 15 cm walls) and two open arms of the same size which crossed in a central-open area of 5 x 5 cm; the maze was located 40 cm above the floor. At the beginning of each trial the mouse was placed in the center of the maze facing one of the open arms. Number of entries and percentage of time spent in the open and closed arms were analyzed for 5 minutes [75]. An entry into an arm occurred when all the four paws of the animal had entered the arm [76]. Before starting and between animals the maze was cleaned with 70% ethanol solution. Data were acquired with a camera installed above the maze, connected to the video-tracking software Ethovision XT 14 (Noldus Information Technology, The Netherlands).

### Y Maze test

The Y maze apparatus consisted of 3 arms of 30 x 5 cm each that were connected to a center zone; height of walls was 15 cm. At the beginning of each trial the mouse was placed in the center of the maze and exploratory activity was measured for 5 minutes. Data were acquired with a camera installed above the maze and the number of entries and spontaneous alternations were manually analyzed offline. An alternation was defined as three consecutive entries into each of the three arms (for example as in any of the following sequences: ABC, CBA, BCA and so on, where each arm was labelled as A, B, or C). An entry into an arm occurred when all the four paws of the animal had entered the arm [76]. The percentage of spontaneous alternations with respect to the maximum possible alternations was calculated as follows: % Alternation= (number of alternations/total number of arm entries - 2) x 100 [77, 78]. Before starting and between animals the maze was cleaned with 70% ethanol solution.

### DLB test

The DLB equipment was made of two chambers of equal size (35 x 30 x 35 cm) connected by a door. One chamber (the light box, LB) was illuminated by a lamp placed above it and had transparent opaque walls, while the other one was with opaque black walls to protect from light and keep that compartment (the dark box, DB) in the darkness. At the beginning of each trial the mouse was placed in the DB and allowed to freely explore the chambers for 5 minutes. The first latency to enter the LB as well as the time spent in that compartment were quantified manually (all the four paws had to be into the LB to consider it as an entry [76]). Before starting and between animals the apparatus was cleaned with 70% ethanol solution.

### Rotarod test

To perform this test we used the mouse Rotarod from Ugo Basile (Varese, Italy). In each trial the mice were placed on the rotating drums and the latency to fall was recorded. Each session consisted of three trials with a 30-min rest interval between each. In the first and second trials the mouse was given a 60- and 20-sec habituation phase, respectively, with the drums rotating at a fixed speed of 4 rpm. In the test phase the Rotarod was set to accelerating mode (from 4 to 40 rpm over 300 sec) and the latency to fall was measured over a period of 5 minutes per each mouse. In the third trial no habituation phase was done. Latency to fall average was calculated for the three trials [21]. Before starting and between each trial the apparatus was cleaned with 70% ethanol solution.

### NOR test

The arena used for the NOR test consisted of a 35 x 35 x 40 cm box with white floor. On Day 1, the mice were placed in the middle of the OF arena for a 5-min habituation trial to freely explore the area. On day 2, two identical objects (oval shape, 6 cm wide x 5 cm high, light red color) were placed in opposite quadrants of the arena and the mice were allowed to freely explore for 10 minutes. On day 3 (test day), one of the familiar objects was replaced with a novel one (conic shape, 4 cm wide x 8 cm high, blue color) and mice were allowed again to freely explore the arena for 10 minutes. Before starting each session and between animals the arena was cleaned with 70% ethanol solution. During the test day, the time spent in exploring the objects was analyzed and discrimination index (DI) was calculated as: DI = (T_N_ − T_F_)/ (T_N_ + T_F_), where T_N_ is exploration time on novel object and T_F_ is exploration time on familiar object. Exploration time was defined as the time when the mouse's nose was pointed towards the object within 2 cm of distance from it. Any time spent sitting on the object without indication of active exploration was not counted as exploration time. Data were acquired with a camera installed above the box, connected to the video-tracking software Ethovision XT 14.

### Contextual FC test

This task was performed in a rectangular shape chamber with transparent walls and a stainless-steel grid floor which was enclosed in a soundproof apparatus (Campden Instruments LTD, England). The conditioning chamber was cleaned with 70% ethanol solution before starting each session and between animals. On day 1, the mice were allowed to explore the context for 2 minutes (habituation phase) and subsequently exposed to three successive mild foot shocks (2 sec duration, 0.3 mA) with 50-sec interval between each. To assess contextual fear memory mice were returned to the same context on day 2 (after 24 h) for a period of 3 minutes. No shock was delivered in this session. Freezing behavior, defined as complete absence of mobility (apart from breathing) within the same area for a time > 2 seconds, was measured through Motor Monitor software (Kinder Scientific).

### Immunoblotting analysis

Western blot experiments were performed on hippocampal tissues which were lysed, and protein levels quantified as previously described [79]. Equal amount of proteins (15 μg) was separated by gel electrophoresis on acrylamide gels (gradient 10-7.5%) and then transferred to a nitrocellulose membrane (Amersham™ Protran®, GE Healtcare). Membranes were milk- or BSA-blocked and then incubated overnight at 4 °C with the following primary antibodies: rabbit anti-phospho N-Methyl-D- aspartate (NMDA) receptor 2A (1:250, Abcam, UK), mouse anti- NMDA receptor 1 (1:2000, BD Bioscience, UK), mouse anti-postsynaptic density protein 95 (PSD95) (1:1000, Abcam, UK) and mouse anti-alpha-tubulin (1:30000; Sigma-Aldrich, USA). Secondary antibody incubations were done for 2 hours at room temperature with anti-rabbit or anti-mouse immunoglobulin G (IgG) at 1:10000 dilutions (LI-COR Biosciences GmbH, Germany). Immunoreactivity was detected by infrared fluorescence with LI-COR® Odyssey® system (LI-COR Biosciences, USA) and quantified using ImageJ 1.48v software (NIH, MA, USA) by densitometry analysis of the immunoreactive bands.

### Blood analysis

Trunk blood was collected postmortem and allowed to clot for 30 min, followed by *3000* *g* centrifugation for 10 minutes at 4 °C to collect the serum fraction [80]. Serum levels of creatinine, albumin and ALT were analyzed using the following assay kits, respectively: ab65340 (Abcam), ab207620 (Abcam) and MAK052 (Sigma-Aldrich). Assays were run according to manufacturer instructions. Some of the serum material was necessary for the optimization of the assays; because of that it wasn’t possible to perform the final tests on the entire number of samples, but we used instead 4-5 samples per group.

### Statistical analysis

The researcher conducting the experiments was blind to control or polypharmacy treatment groups. Data are expressed as mean ± standard error of the mean (SEM), with n indicating the number of animals. Statistical analyses were performed with GraphPad Prism 7 software (San Diego, CA, USA). When comparing two groups, t-Student or Mann-Whitney tests were used for parametric and non-parametric data respectively. Data distribution was assessed with Shapiro-Wilk test. Two-way ANOVA repeated measurements followed by Tukey´s multiple comparison test was used to analyze data when two independent variables were present. A P value ≤ 0.05 was considered as index of significance.
